# Assemblages of saproxylic beetles on large downed trunks of oak

**DOI:** 10.1002/ece3.1935

**Published:** 2016-02-12

**Authors:** Per Milberg, Karl‐Olof Bergman, Kerem Sancak, Nicklas Jansson

**Affiliations:** ^1^ IFM BiologyConservation Ecology GroupLinköping UniversitySE‐581 83LinköpingSweden

**Keywords:** Coleoptera, log, *Quercus robur*, snag, Sweden, veteran tree

## Abstract

Old living oaks (*Quercus robur*) are known as a very species‐rich habitat for saproxylic beetles, but it is less clear to what extent such veteran trees differ from an even rarer feature: downed trunks of large oaks. In this study, we set out to sample this habitat, using window traps, with two aims: (1) to describe the variation of assemblages among downed trunks of different type and (2) to compare beetles on downed oaks with data from veteran standing trees. The results showed that trunk volume and sun exposure better explained assemblages as well as species numbers on downed trunks than did decay stage. Furthermore, species classified as facultative saproxylic species showed weak or no differentiation among downed trunks. Species with different feeding habits showed no apparent differentiation among downed trunks. Furthermore, species composition on dead, downed oak trunks differed sharply from that of living, veteran oaks. Wood or bark feeders were more common on veterans than downed trunks, but there was no difference for those species feeding on fungi or those feeding on insects and their remains. In conclusion, for a successful conservation of the saproxylic beetle fauna it is important to keep downed oak trunks, and particularly large ones, in forest and pastures as they constitute a saproxylic habitat that differs from that of living trees.

## Introduction

Given the large amounts of timber in unlogged forests, it comes as no surprise that a large number of species have more or less specialized on the habitat created by dead and dying timber (Schiegg 2001; Grove 2002). It is also unsurprising that this group is negatively affected by forestry (Hagan and Grove 1999; Komonen et al. 2008), as it severely reduces the available habitat (Speight 1989; Andersson and Hytteborn 1991; Linder and Östlund 1998; Sippola et al. 1998; Schiegg 2000; Davies et al. 2007; Bishop et al. 2009; Seibold et al. 2015). The saproxylic insect fauna is, during some part of their life cycle, dependent on the dead or dying wood of moribund or dead trees, or upon wood‐inhabiting fungi, or upon the presence of other saproxylic species (Speight 1989). It is somewhat of an enigma that so many saproxylic species can utilize a habitat that is so spatially confined, and relatively uniform, as dead wood. Still, looking at the finer details, it is known that saproxylic assemblages can vary between tree species (Milberg et al. 2014) and depending on, for example, factors such as whether the wood decay occurs outside of the tree (trunk and branches) or inside the tree in hollow (Winter and Möller 2008; Jansson et al. 2009a; Quinto et al. 2014), if the wood is living or dead standing (Jonsell and Weslien 2003), standing or downed (Franc 2007; Andersson et al. 2015), and burned or not (Toivanen and Kotiaho 2007). Apart from these attributes, degree of decay, trunk size, level of sun exposure, wood decaying fungi colonization have also been pointed out as important variables for saproxylic species assemblages (Hjältén et al. 2007; Brin et al. 2011; Bouget et al. 2013). A recent study pointed out dead wood of large diameter, dead wood of broad‐leaved trees, and dead wood in sunny areas as particularly important for rare and red‐listed species in central Europe (Seibold et al. 2015). A main candidate for such a timber in Europe is veteran oaks (i.e., very large and old oaks) that are uniquely rich in saproxylic species, many of which are considered as oak specialists (preferentially using oak), or even considered as confined to oaks (but see Milberg et al. 2014).

As studies comparing standing and downed timber have pointed to a difference in species composition (e.g., Hammond et al. 2001, 2004; Jonsell and Weslien 2003; Lindhe et al. 2004; Wikars et al. 2005; Gibb et al. 2006; Hjältén et al. 2007, 2012; Fossestøl and Sverdrup‐Thygeson 2009; Ulyshen and Hanula 2009; Ulyshen et al. 2011; Brin et al. 2013; Andersson et al. 2015), there is ground to assume downed veteran oak timber as a unique habitat with a high incidence of rare species. Furthermore, the volume of downed oak timber is quite modest compared with the volume of living oaks in Swedish forests (Nordén et al. 2004b; personal observations). So far, the knowledge about downed oak as a saproxylic habitat is small (Franc 2007; Bouget et al. 2012), compared with living oaks (e.g., Buse et al. 2008; Bergman et al. 2012; Horak et al. 2014) or downed trunks of other tree species (e.g., Bunnell and Houde 2010; Müller and Bütler 2010; Lassauce et al. 2011). Franc (2007) examined saproxylic beetles in 13 forests in Sweden and noted striking differences in assemblages between the lying and standing dead wood of oak. Lying dead wood had more fungivores and fewer primary and secondary wood boring species but did not differ in number of red‐listed species (Franc 2007). Bouget et al. (2012) sampled saproxylic beetles assemblages on lying and standing dead oak logs in a forest in France and also found differing assemblages, and a slight difference among feeding guilds. However, in contrast to Franc (2007), Bouget et al. (2012) reported higher richness for standing than lying dead wood.

In the present study, we sampled 40 large, downed oak trunks with window traps in one of the most oak‐rich regions in Sweden (Musa et al. 2013). As species richness is known to increase with trunk size (e.g., Ranius and Jansson 2000; Grove and Forster 2011), we aimed for trunks that were substantially larger than those sampled by Franc (2007; window traps) and Bouget et al. (2012; emergence traps). We used window traps due to the large size of the trunks that made emergence traps less feasible. The species‐rich data collected were used (1) to compare saproxylic species composition among large downed oaks differing in trunk characteristics – decay stage, trunk volume, sun exposure – and (2) to compare saproxylic beetle species composition in large downed oaks with existing data from a selection of large standing, veteran oaks.

We expected obligate saproxylic species to be more clearly differentiated among downed logs than facultative saproxylic species (the latter by definition being feeding generalists). Among the three characteristics of downed logs, we expected stage of decomposition to be most important as a high score represent a greater variety of stages in the succession of decomposition (Pyle and Brown 1999), hence allowing for successional niche differentiation. Then, we expected sun exposure to be relatively important, as it suggests microclimate differentiation (with least variation in shaded logs). Finally, we expected log volume to be least important in term of allowing for niche separation between saproxylic species; it simply reflects the amount of resource, but no differentiation in terms of feeding niches.

Furthermore, we expected a difference between downed and standing oaks that could be explained by feeding guilds: bark and wood eaters would be most prevalent on living trees while fungus eaters would be most prevalent on downed trees (due to moist conditions and larger volume of decomposing wood; Franc 2007; Bouget et al. 2012; Andersson et al. 2015). For predators, we did not expect a difference (as in Bouget et al. 2012), unless there was a substantial difference in abundance (i.e., more food).

## Material and Methods

### Study sites

The study was conducted southeast of Linköping in the County of Östergötland in Sweden. The Swedish Environmental Protection Agency has identified this region in Sweden as particularly valuable from a conservation point of view (Lindbladh et al. 2007; Claesson and Ek 2009). The region consists mainly of pastures, arable fields, mixed and conifer forests, and lakes, and its distinguishing feature is the large number of large oaks. In the study area, we selected six sites: Bjärka Säby, Bjärka Storäng, Grebo, Brokind, Sturefors, and Tinnerö. All of them have a high oak density (Claesson and Ek 2009) and are known to have a rich saproxylic beetle fauna (Ranius and Jansson 2000, 2002; Andersson et al. 2014).

### Sampling method

**Figure 1:**
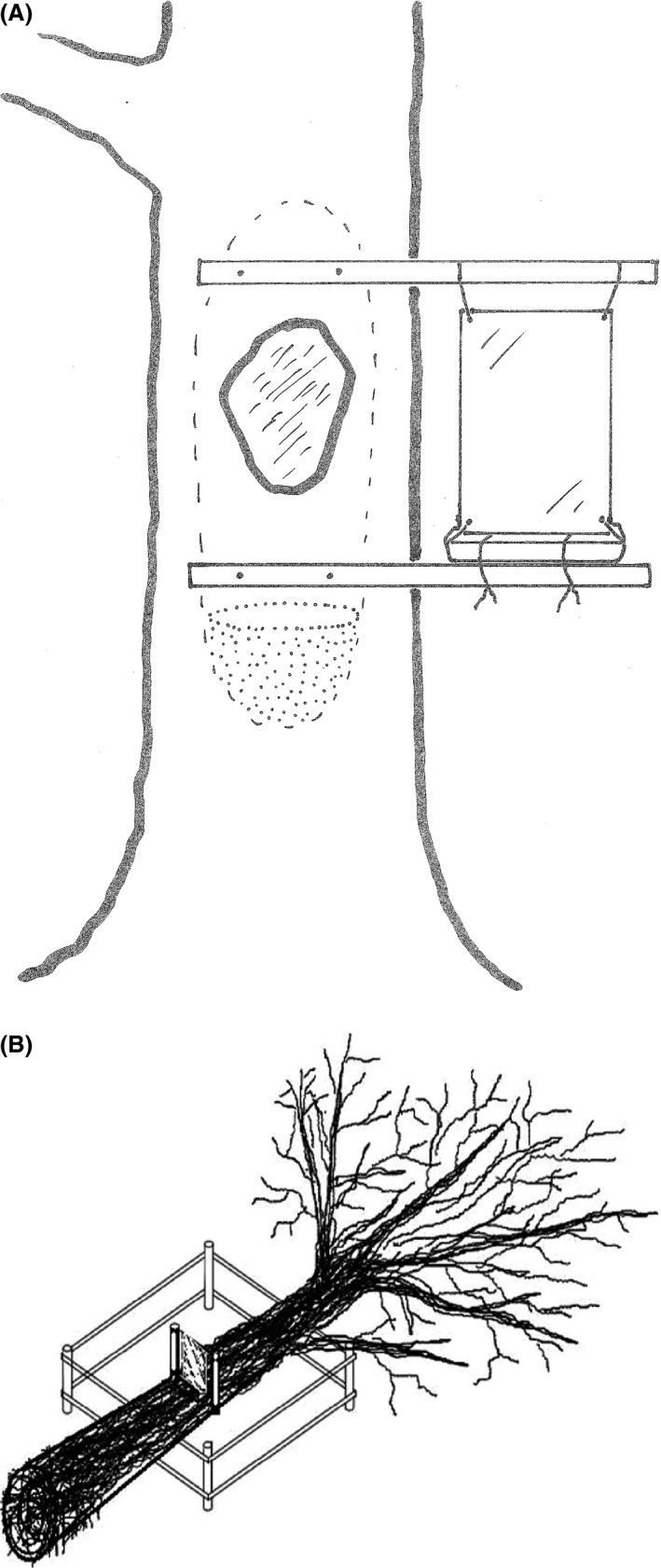
Sketch of a window trap mounted on a living oak (A), and on a downed oak (B).

Saproxylic beetles were collected using window traps. The window trap consisted of a 30 cm × 50 cm‐wide transparent plastic plate with an aluminum tray underneath. The tray was filled with equal amounts of propylene glycol and water, and 0.25 L of denatured alcohol was added to prevent grazing animals from drinking the solution. A number of drops of a washing detergent were then added to eliminate surface tension. The traps were mounted in early May and emptied once a month, until late August (Table 1). In total, 40 downed oaks were sampled in 2009 and 2010. Data from a matched number of living large hollow oaks from the same sites were selected from trees that had been sampled with window traps in 1994 and 2008 (Table 1). These oaks were initially selected to represent a broad spectrum of the best saproxylic environments at sites, that is, large and old trees with many and/or large hollows and with different types of rot (Jansson et al. 2009b; Bergman et al. 2012). Large logs were searched and selected with the aim of attaining variation within each site in the three log attributes under study: decay stage, sun exposure and log volume. Another criterion for selection was to avoid logs with living hollow oaks within 20 m (the rarity of large logs prevented this criteria from being fulfilled at some sites). There was one trap per log placed either in the middle of logs that had few branches remaining (i.e., old and decayed logs) or more toward the base on younger logs with branches remaining (as the latter could obscure the flight path of insects; Fig. 1B). The window trap was always at right angle with the log direction (Fig. 1B). As the downed oaks were mainly situated in wooded pastures, window traps were fenced with a wooden 4 m × 4 m construction to exclude grazing animals (Fig. 1B).

**Table 1:** Study sites, number of sampled oak (*Quercus robur*) trees and downed logs with sampling years

Study site	*N*	Large downed logs	Veteran standing trees
		Sampling year	Sampling year
Tinnerö	10	2010	2008
Bjärka Säby	10	2010	1994
Brokind	5	2010	1994
Sturefors	5	2010	1994
Bjärka Storäng	5	2009	1994
Grebo	5	2009	1994

For the downed oak logs, records of the three target variables were noted: decay stage, log volume, and sun exposure (Table 2). Decay stage classification systems have been developed for coniferous timber (McCullough 1948; Lindblad 1998) and modified for deciduous timber (Ódor and van Hees 2004), and are based on cover of bark, presence of twigs and branches, softness of the wood, and the surface and shape of the log. However, among the very large oak trunks involved our study there were several that had substantial internal decay developed before being downed, hence being completely hollow but still very hard on the outside (cf. Grove et al. 2011). To account for such a combination of characteristics, we developed a simplified system for large downed oak trunks that had four classes: (1) bark stable and hard to break off; (2) with at least some bark, and that can be broken off while wood is still hard; (3) no bark, and wood at least partly rotten and with red or yellow color; and (4) no bark, and wood rotten with dark color, and to great depth; or completely hollow. To estimate volume and sun exposure, the trunk was first considered as comprised of a number of truncated cones (called segments) that each were relatively uniform in shape. Most trunks (31 of 40) had 2‐4 such segments, while three had more than seven. For each segment, its length and circumference at the two ends were measured. For volume, we then calculated the sum of volume of all segments. We estimated the percent of the segment that was directly sun‐exposed, and calculated the surface area of the top half of the segment. These two segment attributes were then used to calculate a weighted average of “sun exposure” for the trunk. These three variables turned out to be relatively uncorrelated (*R* ^2 ^= 0.027, 0.036, and 0.089).

**Table 2:** Characteristics of sampled downed trunks of oak (*Quercus robur*) and living trees used for comparisons. Sun exposure was the area‐weighted average of several estimates; decay stage was scored as one of four ordinal classes

	Average	SD	Minimum	Maximum
Downed trunks (*N* = 40)				
Largest diameter (cm)	73 cm	22	38	115
Volume (m^3^)	3.4	2.9	0.45	14.2
Sun exposure (%)	55.5	21.6	5.0	90.0
Decay stage	1.9	1.0	1	4
Living trees (*N* = 39)^a^				
Diameter at breast height (cm)	109	25	60	174

a Data are missing from one of the 40 trees used.

### Species identification

The saproxylic beetles from downed oaks were identified to species level by Nicklas Jansson, Stig Lundberg, and Rickard Andersson. The taxonomic ambition was lower for the material collected from standing trees (where a number of families had been left out due to lack of time). More specifically, it concerned the following saproxylic taxa that were not identified: Latrididae, Ptiliidae, Leiodidae, Melyridae, Monotomidae, Mordellidae, Nitidulidae, Pyrochroidae, Salpingidae, Scirtidae, Scolytidae, Scraptidae (except genus *Scraptia*), and Staphylinidae (except genera *Velleius, Quedius*, and former family Pselaphidae). Hence, they were excluded from the data from downed trunks when these were compared with data from living trees.

Identified species were classified as facultative and obligate saproxylic – indicating their degree of association with deadwood – according to the saproxylic database (Dahlberg and Stokland 2004; Stokland and Meyke 2008). Identified species were also assigned to feeding guilds based on Palm (1959), considering three types of food sources: (1) wood or bark, (2) fungi, and (3) insects and their remains. N.B. only two‐thirds of the species identified were classified and there are a few cases were a species belonged to more than one feeding guild.

Nomenclature follows Lundberg (1995).

### Data analysis

The species‐rich data from downed trunks were primarily analyzed from the point of view of composition of species, as that might carry more information than simple variables like species richness. Hence, the log_10_(*x* + 1)‐transformed species data were subjected to two multivariate analyses: one with facultative saproxylic species (*N* = 115) and one with obligate saproxylic species (*N* = 202). We used the software CANOCO 4.5 (ter Braak and Šmilauer 1998), and partial constrained analyses. A constrained ordination means considering only the variation in species composition that a set of explanatory variables can explain, while a partial ordination means eliminating the effect of a covariable (Šmilauer and Lepš 2014). In our case, the six sites were used as categorical covariables, hence, adjusting for site differences. The explanatory variables considered were (1) decay stage, (2) trunk volume, and (3) sun exposure, one by one as well as combined, and their explanatory power evaluated in permutations (*N* = 9999), with permutation blocks defined by sites. As beta diversity (turnover of species from one trap to the next) in data was modest (3.5 and 2.1 SD units for facultative and obligate species, respectively), we chose partial redundancy analyses (pRDA). In addition, generalized linear models (normal distribution and identity link) were used to predict number of species (obligate and facultative species separately) using “site,” decay stage, trunk volume, and sun exposure as explanatory variables.

Data from standing and downed trees were contrasted using odds ratios, that is, the ratio between the odds of finding a species on the 40 living trees and the corresponding odds for the 40 downed trunks. These specieswise odds ratios were then subjected to metaanalysis, using the software Comprehensive Metaanalysis 2.0 (Borenstein et al. 2005) to calculate: (1) a weighted average for species classified as obligate and facultative saproxylic species; (2) and weighted averages for per feeding guild. Number of specimens per trap (ln‐transformed) and number of species per trap were analyzed with ANOVA.

## Results

### Downed oaks

In total, we identified 7 745 specimens of 317 species from the 40 downed oaks (Table 3; Appendix S1).

**Table 3:** Characteristics of the saproxylic beetles sampled in downed logs of oak (*Quercus robur*). Species are classified as obligate or facultative saproxylic

	Downed logs (*N* = 40)	±CI_95%_
Obligate saproxylic		
Total specimens	4751	
Total species	202	
Singletons^a^	51	
Red‐listed species	24	
Specimens per trap	118.8	18.13
Species per trap	34.0	3.51
Facultative saproxylic		
Total specimens	2994	
Total species	115	
Singletons^a^	25	
Red‐listed species	0	
Specimens per trap	74.8	16.05
Species per trap	18.7	1.94

a Number of species occurring with only one specimen.

Species composition of obligate saproxylic species was clearly differentiated among downed logs (pRDA with decay stage, trunk volume, and sun exposure as explanatory variables; permutation test *P* = 0.0006). In contrast, the composition of facultative saproxylic species could not be explained by trunk characteristics (*P* = 0.8208) and were therefore not considered in more detail.

**Figure 2:**
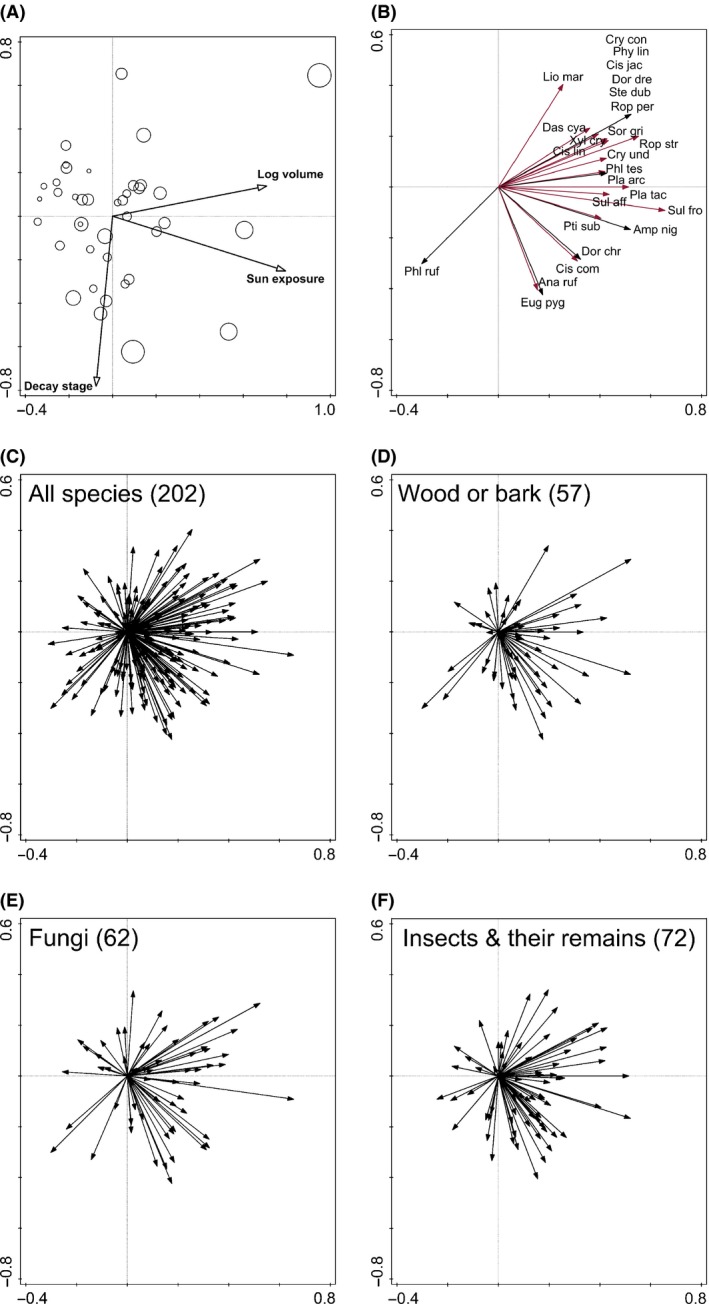
Partial redundancy analyses of obligate saproxylic beetle data from downed oaks and their relationship with environmental variables, after covarying out the site effect. (A) The explanatory variables (arrows) with individual tree trunks indicated by circles which size is proportional to species richness. (B) The 25 species that were best explained by the three explanatory variables. (C) Each species represented by an arrow. (D–F) Species selected according to feeding guild (indicated in graph, together with number of species in guild).

In the pRDA of the obligate saproxylic species, the most important explanatory variables were sun exposure and trunk volume while decay stage seemed least important (Fig. 2A, Table 4). Species associated with large/sun‐exposed logs were, for example, *Ropalodontus strandi, Sulcacis fronticornis, Ampedus nigroflavus* while *Anaspis rufilabris, Euglenes pygmaeus/oculatus,* and *Phloiotrya rufipes* were associated with late decay stages (Fig. 2B).

**Table 4:** How the three attributes of downed oak (*Quercus robur*) logs explain species composition among obligate saproxylic species according to pRDA (using the six study sites as covariables). *P*‐values emerged from permutation tests with 9999 permutations, using the sites as permutation blocks

	Explained variance (%)	*P*
Decay stage	4.1	0.0906
Trunk volume	5.3	0.0323
Sun exposure	5.4	0.001
Decay stage & Trunk volume	9.6	0.0089
Decay stage & Sun exposure	9.6	0.0009
Trunk volume & Sun exposure	9.9	0.0022
Decay stage & Trunk volume & Sun exposure	14.2	0.0006

In the pRDA of obligate saproxylic species (Fig. 2), we expected the substantial differentiation among trunks due to decay stage, volume, and sun exposure to coincide with different feeding guilds. This, however, was not the case as there was no apparent difference in the multivariate differentiation among guilds (lengths and directions of arrows were more or less similar in Fig. 2C–F). This lack of differentiation was also confirmed in MANOVA using the three guilds to explain the specieswise ordination results for the constrained ordination axes (data not shown).

The number of species of obligate saproxylic species increased with trunk volume, sun exposure, and decay stage (Table 5). Patterns were much weaker or nonexistent among the facultative beetles, and only sun exposure could explain part of the variation in species numbers recorded (Table 5).

**Table 5:** How number of species is explained by three trunk characteristics of downed oaks (*Quercus robur*) and site (treated as six categories). Generalized linear model (normal distribution; identity link) was used, and in all cases, slope estimates for decay stage, sun exposure, and trunk volume were positive

	Obligate saproxylic		Facultative saproxylic	
	Wald	*P*	Wald	*P*
Decay stage	8.26	0.0040**	1.37	0.242
Sun exposure	19.3	<0.0001***	4.16	0.0413*
Trunk volume	24.3	<0.0001***	0.417	0.518
Site	39.8	<0.0001***	32.1	0.0001***

*P < 0.05, **P > 0.01, ***P > 0.001.

### Downed oaks versus living oaks

In total, this comparison involved 3 273 specimens of 130 species (Appendix S2). For the obligate species, downed logs had less than half the total number of specimens but despite this almost twice the total number of species compared with standing trees (Table 6). In contrast, the facultative species were much more numerous on downed logs but nevertheless did not differ much in number of species (Table 6). The proportion of red‐listed species was somewhat higher among downed trunks than living trees (Table 6).

**Table 6:** Characteristics of the saproxylic beetle fauna recorded in the data used to compare downed logs with standing living trees of oak (*Quercus robur*). Species are classified as obligate or facultative saproxylic

	Downed logs (*N* = 40)	Trees (*N* = 40)	*F*_(1,73)_ b	*P*
Obligate saproxylic				
Total specimens	1043	2230		
Total species	110	67		
Species unique to Downed logs or Trees	63	20		
Singletons^a^	34	17		
Red‐listed species	11	4		
Specimens per trap (±CI_95%_)	26.1 (5.85)	55.8^c^ (43.98)	0.782	0.379
Species per trap (±CI_95%_)	12.9 (1.73)	11.2 (1.66)	1.83	0.180
Facultative saproxylic				
Total specimens	782	212		
Total species	34	29		
Species unique to Downed logs or Tree	17	13		
Singletons^a^	9	10		
Red‐listed species	0	0		
Specimens per trap (±CI_95%_)	19.6 (4.30)	5.3 (1.51)	47.1	<0.0001
Species per trap (±CI_95%_)	5.9 (0.80)	2.9 (0.55)	37.8	<0.0001

a Number of species occurring with only one specimen.

b Sites were considered as a block factor; number of specimens were ln‐transformed.

c 34.3 (±13.31) if excluding an outlier.

Catch per trap varied substantially, but did not differ significantly between downed logs and standing trees for the obligate species (Table 6). In contrast, there were many more specimens and twice the number species of facultative species in traps on downed logs (Table 6).

**Figure 3:**
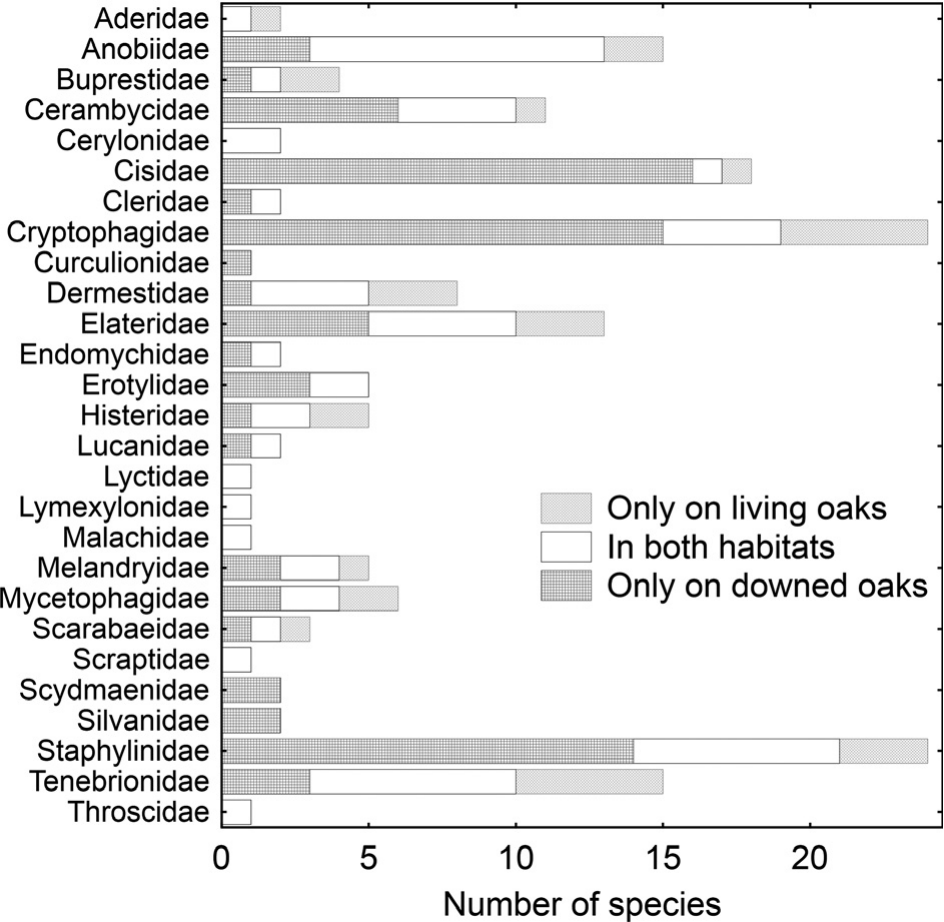
Number of species of some key families recorded and their distribution among downed trunks and standing trees.

Beta diversity (total number of species divided by average number per trap) was higher for obligate species on downed logs than among standing trees; the reverse was true for the facultative species. Three families of insects were only recorded in traps on downed trunks (Curculionidae, Scydmaenidae, Silvanidae), while none were exclusive for the living trees (Fig. 3). Furthermore, some families were more species‐rich on downed trunks than on living trees (e.g., Cryptophagidae, Cisidae, and Cerambycidae; Fig. 3).

**Figure 4:**
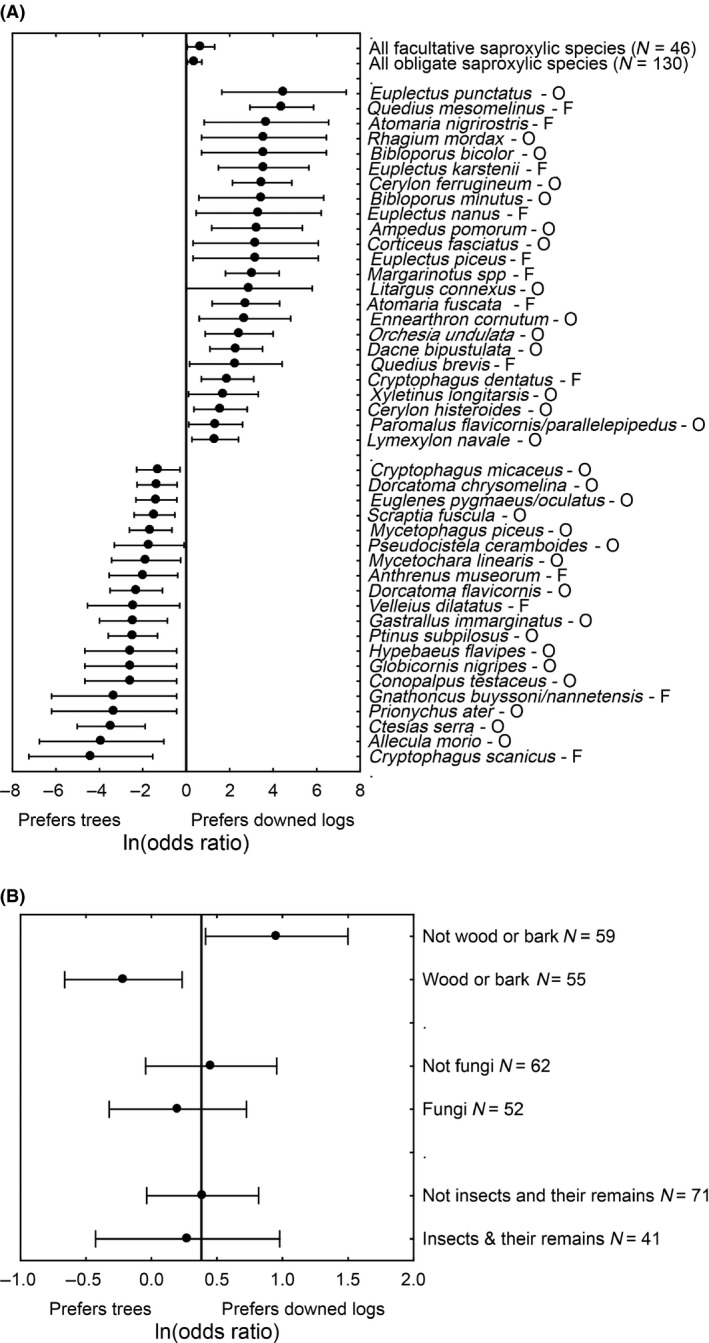
The preferential occurrence of saproxylic beetles on downed trunks and standing trees of oak. Graph show ln(odds ratio) for (A) those species with a significant association, and (B) the weighted average per feeding guild (vertical line indicate the overall average preference of species).

According to the meta analysis both obligate and facultative saproxylic species showed a overall preference for downed trunks, but the degree of preference did not differ between them (Fig. 4A). Of the 130 species, 44 showed a significant affiliation with either downed trunks (*N* = 24) or standing trees (*N* = 20) (Fig. 4A). Among the species for which feeding guilds were available, there was a very clear difference among wood or bark feeders that were much more frequent on standing trees than downed logs (Fig. 4B). There were no clear differences for those feeding on fungi or those feeding on insects and their remains (Fig. 4B).

## Discussion

### There is differentiation among downed oaks

An important new finding in our study is that there can be substantial differences in beetle assemblages among large downed trunks, adding to the previously established importance of trunk size (Bouget et al. 2012). It is noteworthy that this differentiation only concerned the obligate saproxylic species, while the facultative ones – that are more of feeding generalists – seemed unaffected by trunk characteristics considered in our study (cf. Gough et al. 2015). This confirmed our initial expectation and suggests that the occurrence of facultative saproxylic species is mainly determined by other factors than the trunk attributes considered here.

Among the three characteristics of downed logs considered in the present study, we expected stage of decomposition to be most important as a high score represent a greater variety of stages in the succession of decomposition (Grove et al. 2011) and hence allows for successional niche differentiation. Then, we expected sun exposure to be relatively important, as it implies differentiation in microclimate (with least variation in shaded logs). Finally, we expected log volume to be least important in term of allowing for niche separation between saproxylic species. Our assumptions were not confirmed. Rather, decay stage seemed least important, while sun exposure and trunk volume were most important. Two of the species with high preference for sun‐exposed logs in our study were also classified as more common in sunny conditions by Palm (1959; *Ampedus nigroflavus* and *Plagionotus arcuatus*). Whether sun exposure is a positive or negative feature for the saproxylic diversity of a log is likely to be climate‐dependent, being negative in warm and dry climates (Quinto et al. 2014) and positive in humid ones (the current study; Ranius and Jansson 2002).

It is perhaps not surprising that trunk volume was so important differentiating among trunks (e.g., Jacobs et al. 2007; Bishop et al. 2009), as it corresponds to the amount of available habitat. Furthermore, larger wood volume also takes longer to decompose (allowing for the accumulation of species) and provides more heterogeneous habitats for saproxylic beetles than smaller wood volume (Grove 2002). Nor is it perhaps surprising that sun exposure is so important, as it is likely to affect the temperature of the wood as well as its moisture content, and has been shown important for other tree species in several situations (Sverdrup‐Thygeson and Ims 2002; Jonsell et al. 2004; Brunet and Isacsson 2008; Fossestøl and Sverdrup‐Thygeson 2009). Together, this suggests that differentiation according to decomposition succession is less important than niche differentiation according to microhabitat conditions, a suggestion with implications for the “saproxylic enigma,” that is, the coexistence of a large number of species in a confined and relatively homogeneous habitat. It will be interesting to see if our findings – based on window‐trapped material – are confirmed when using emergence traps.

### Downed oaks and living oaks differ

Our study clearly confirms that downed trunks and standing trees of the same species can differ greatly in their composition of saproxylic species (e.g., Gibb et al. 2006; Fossestøl and Sverdrup‐Thygeson 2009; Ulyshen and Hanula 2009; Hjältén et al. 2012; Brin et al. 2013; Andersson et al. 2015). By being one of few studies of oaks, it also confirmed the finding by Franc (2007) and Bouget et al. (2012). In our study, however, we found most species and most rare species on downed trunks while Bouget et al. (2012) found most on their standing dead wood; Franc (2007) found no difference. Whether this is due to a more depauperate fauna in the production forest in the study by Bouget et al. (2012), the differing methods used (emergence vs. window traps), to our trunks being much larger, or to us contrasting downed wood with living trees, remains unclear. The fact that the conclusions regarding differences differ is, however, far from surprising given the conflicting results in the literature (e.g., Jonsell and Weslien 2003; Hammond et al. 2004; Lindhe et al. 2004; Wikars et al. 2005; Gibb et al. 2006; Hjältén et al. 2007, 2012; Ulyshen and Hanula 2009). Hence, at this point we can conclude that our large, downed trunks have higher conservation value for saproxylic species than large, veteran oaks, but the transferability of this finding is modest as it does not apply over all situations. It is also worth noting that living veteran trees probably support more nonsaproxylic species than downed trunks.

It is always difficult to compare magnitude of dissimilarity among studies (and also within our study as sampling was performed in different years). Nevertheless, given that differences in saproxylic assemblages among tree species can be quite modest (Milberg et al. 2014), the difference documented here between standing and downed wood seems very large. Hence, large trees that die and have fallen down seem to provide a distinctly different habitat for many saproxylic organism than standing living veterans (the present study) or dead, standing trees (Franc 2007; Bouget et al. 2012). Due to their rarity and high proportion of red‐listed species, downed large oaks ought to receive much more consideration in conservation work than hitherto.

How then can we explain this difference among living trees and downed dead wood of large oaks? We found differences in occurrence related to feeding guilds, but not according to the broader grouping as facultative and obligate saproxylic species. Regarding feeding guilds, we had some initial expectations. First, we expected that bark and wood feeders would be most prevalent on living trees as they are alive, and this assumption was confirmed. Second, we had expected those feeding on fungi to be more prevalent on downed trees due to their more moist condition (Franc 2007; Bouget et al. 2012; Andersson et al. 2015) and also because of their larger total volume of decomposing wood compared with living standing veteran trees. This assumption was not confirmed, so the simplistic assumption of more fungal food for beetles in downed trunks than standing veterans might not hold. It is possible that a more diverse or specialist fungal flora of dying or newly dead wood compared with decomposed wood (van der Wal et al. 2015) provide an ample food source for beetles, or a more diverse one that allows for feeding specialists. Living veterans might also carry more fine woody material (finer branches and twigs) than partly decomposed downed logs, adding to a differing fungal composition (Nordén et al. 2004a,b). Third, we confirmed our last assumption, that there was no pattern among the beetle species feeding on insects and their remains (cf. Bouget et al. 2012).

Among the species with a strong preference for either standing or downed oaks we found a higher degree of obligate saproxylic species with preference for standing trees. One explanation can be the position of the microhabitats and the traps. The traps on downed trees were situated 0.5–1 m above ground level while on living trees were situated 2–5 m from ground. This means that some of the species (e.g., *Cryptophagus dentatus, Atomaria fuscata*, and *Margarinotus* spp.) caught in the traps on downed trees can also originate from the litter, that is, leaves in compostlike situations.

Many of the species with a strong preference for standing hollow oaks required microhabitats for larval development in cavities in the trunk, for example, *Allecula morio, Gnathoncus buyssoni/nannetensis, Prionychus ater, Pseudocistela ceramboides, Mycetochara linearis, Globicornis nigripes, Hypebaeus flavipes*, and *Ptinus subpilosus* (Ranius and Jansson 2002). Also some species living on red‐rotted wood on the trunk, like *Mycetophagus piceus, Dorcatoma flavicornis,* and *Dorcatoma chrysomelina* (Hansen 1951; Palm 1959), were more common on standing trees.

The species with a strong preference for the downed oaks were often species living in fruit bodies of fungi or fungal‐infested wood (e.g., *Ennearthron cornutum, Dacne bipustulata Hypylus quercinus*, and *Orchesia undulate*) or species living under moist bark (*Litargus connexus, Euplectus punctatus, Bibloporus minutus, Bibloporus bicolor, Euplectus piceus, Euplectus karsteni, Cerylon histeroides,* and *Cerylon ferrugineum*; Hansen 1951; Palm 1959). The latter five species have also shown similar patterns in a previous oak study (Franc 2007).

### Conservation implications

First, large downed trunks carry other parts of biodiversity than standing veteran oaks, so it might be appropriate to consider downed veteran trees as a saproxylic environment distinctly different from living veteran trees. This means that enrichment of dead wood, that is, when dead trunks are placed on the ground to enhance the saproxylic value of sites (Gossner et al. 2013; Floren et al. 2014), might have its greatest conservation value by providing a different habitat, rather supplementing that of standing trees. Second, as large downed trunks are much rarer than veteran trees, they should be given special attention, especially as downed trunks seem richer in species and with more rare species than living trees. There is, however, not evidence enough to suggest felling veteran oaks to create downed trunks. Third, as downed trunks can be quite different in their composition of saproxylic species, their diversity is likely to increase with intertrunk differences in placement in the field. At least in the humid climate of the present study sites, the sun‐exposed were the most valuable, suggesting that partial clearing around newly fallen logs might benefit conservation targeting saproxylic environments.

## Conflict of Interest

None declared.

## Supporting information


**Appendix S1.** Saproxylic species recorded in traps placed on downed trunks of large oaks, their red‐list status (according to Gärdenfors 2010), and their frequency in 40 window traps.

**Appendix S2.** Saproxylic species included in the contrast between downed trunks of oaks and living veteran oaks.
