# Supplementary material for: Assemblages of saproxylic beetles on large downed trunks of oak
Source: Ecol Evol. 2016 Feb 12;6(6):1614–25. doi: 10.1002/ece3.1935 (PMC4751154; doi:10.1002/ece3.1935)
Supplement: Supplementary file 1 — Appendix S1. Saproxylic species recorded in traps placed on downed trunks of large oaks, their red‐list status (according to Gärdenfors 2010), and their frequency in 40 window traps. [file ECE3-6-1614-s001.docx]

**Appendix 1.** Saproxylic species recorded in traps placed on downed trunks of large oaks, their red-list status (according to Gärdenfors 2010), and their frequency in 40 window traps.

| **Species** | **Family** | **Redlisted** | **Frequency**  **(max 40)** |
| --- | --- | --- | --- |
| **Obligate saproxylic species** |  |  |  |
| Plegaderus caesus | Histeridae |  | 1 |
| Paromalus flavicornis/parallelepipedus | Histeridae |  | 12 |
| Platysoma deplanatum | Histeridae | NT | 1 |
| Ptenidium turgidum | Ptiliidae |  | 4 |
| Anisotoma humeralis | Leioddidae |  | 39 |
| Anisotoma castanea | Leioddidae |  | 25 |
| Anisotoma glabra | Leioddidae |  | 10 |
| Anisotoma orbicularis | Leioddidae |  | 19 |
| Liodopria serricornis | Leioddidae | NT | 4 |
| Agathidium pisanum | Leioddidae |  | 5 |
| Stenichnus godarti | Scydmaenidae |  | 3 |
| Quedius maurus | Staphylinidae |  | 1 |
| Quedius plagiatus | Staphylinidae |  | 2 |
| Nudobius lentus | Staphylinidae |  | 2 |
| Bibloporus bicolor | Staphylinidae |  | 12 |
| Bibloporus minutus | Staphylinidae |  | 11 |
| Euplectus decipiens | Staphylinidae |  | 2 |
| Euplectus bescidicus | Staphylinidae |  | 3 |
| Euplectus punctatus | Staphylinidae |  | 21 |
| Euplectus mutator | Staphylinidae |  | 3 |
| Batrisodes venustus | Staphylinidae |  | 1 |
| Phyllodrepa linearis | Staphylinidae |  | 1 |
| Phyllodrepa ioptera | Staphylinidae |  | 21 |
| Hapalaraea pygmaea | Staphylinidae |  | 6 |
| Phloeonomus pusillus | Staphylinidae |  | 3 |
| Phloeonomus sjoebergi | Staphylinidae |  | 3 |
| Scaphidium quadrimaculatum | Staphylinidae |  | 1 |
| Scaphisoma subalpinum | Staphylinidae |  | 3 |
| Scaphisoma assimile | Staphylinidae |  | 1 |
| Carphacis striatus | Staphylinidae | VU | 6 |
| Oxypoda arborea | Staphylinidae |  | 5 |
| Phloeopora testacea | Staphylinidae |  | 8 |
| Phloeopora nitidiventris | Staphylinidae |  | 1 |
| Phloeopora corticalis | Staphylinidae |  | 10 |
| Phloeopora concolor | Staphylinidae |  | 2 |
| Atheta castanoptera | Staphylinidae |  | 26 |
| Atheta britanniae | Staphylinidae |  | 23 |
| Dinaraea aequata | Staphylinidae |  | 1 |
| Dinaraea linearis | Staphylinidae |  | 1 |
| Thamiaraea cinnamomea | Staphylinidae |  | 8 |
| Thamiarea hospita | Staphylinidae | NT | 2 |
| Agaricochara latissima | Staphylinidae | NT | 1 |
| Bolitochara mulsanti | Staphylinidae |  | 1 |
| Leptusa pulchella | Staphylinidae |  | 9 |
| Leptusa fumida | Staphylinidae |  | 1 |
| Tachyusida gracilis | Staphylinidae | VU | 2 |
| Anomognathus cuspidatus | Staphylinidae |  | 23 |
| Placusa depressa | Staphylinidae |  | 7 |
| Placusa tachyporoides | Staphylinidae |  | 17 |
| Placusa atrata | Staphylinidae |  | 6 |
| Prionocyphon serricornis | Scirtidae |  | 2 |
| Liocola marmorata | Scarabaeidae |  | 7 |
| Lucanus cervus | Lucanidae |  | 1 |
| Sinodendron cylindricum | Lucanidae |  | 5 |
| Denticollis linearis | Elateridae |  | 26 |
| Ampedus sanguineus | Elateridae |  | 1 |
| Ampedus nigroflavus | Elateridae | NT | 3 |
| Ampedus pomorum | Elateridae |  | 16 |
| Ampedus hjorti | Elateridae |  | 1 |
| Ampedus balteatus | Elateridae |  | 7 |
| Ampedus praeustus | Elateridae |  | 5 |
| Melanotus villosus | Elateridae |  | 2 |
| Melanotus castanipes | Elateridae |  | 6 |
| Cardiophorus ruficollis | Elateridae |  | 1 |
| Microrhagus lepidus | Eucnemidae | NT | 7 |
| Microrhagus pygmaeus | Eucnemidae |  | 5 |
| Agrilus biguttatus | Buprestidae |  | 4 |
| Agrilus sulcicollis | Buprestidae |  | 2 |
| Globicornis emarginata | Dermestidae |  | 1 |
| Globicornis nigripes | Dermestidae | NT | 1 |
| Ctesias serra | Dermestidae |  | 2 |
| Lyctus linearis | Bostrichidae | VU | 1 |
| Ptinus rufipes | Anobiidae |  | 21 |
| Ptinus subpilosus | Anobiidae |  | 5 |
| Hedobia imperalis | Anobiidae |  | 1 |
| Xestobium rufovillosum | Anobiidae |  | 2 |
| Gastrallus immarginatus | Anobiidae |  | 2 |
| Anobium rufipes | Anobiidae |  | 1 |
| Microbregma emarginata | Anobiidae |  | 2 |
| Xyletinus pectinatus | Anobiidae |  | 1 |
| Xyletinus longitarsis | Anobiidae | VU | 9 |
| Dorcatoma flavicornis | Anobiidae |  | 4 |
| Dorcatoma chrysomelina | Anobiidae |  | 14 |
| Dorcatoma dresdensis | Anobiidae |  | 1 |
| Lymexylon navale | Lymexylonidae |  | 16 |
| Tillus elongatus | Cleridae |  | 1 |
| Trichoceble floralis | Melyridae | NT | 1 |
| Dasytes niger | Melyridae |  | 17 |
| Dasytes cyaneus | Melyridae |  | 5 |
| Dasytes plumbeus | Melyridae |  | 39 |
| Hypebaeus flavipes | Malachidae |  | 1 |
| Malachius bipustulatus | Malachidae |  | 7 |
| Epuraea guttata | Nitidulidae |  | 10 |
| Epuraea neglecta | Nitidulidae |  | 4 |
| Epuraea pallescens | Nitidulidae |  | 4 |
| Epuraea marseuli | Nitidulidae |  | 3 |
| Epuraea pygmaea | Nitidulidae |  | 2 |
| Epuraea biguttata | Nitidulidae |  | 2 |
| Epuraea muehli | Nitidulidae |  | 1 |
| Epuraea silacea | Nitidulidae |  | 1 |
| Soronia grisea | Nitidulidae |  | 34 |
| Ipidia binotata | Nitidulidae | NT | 2 |
| Cryptarcha strigata | Nitidulidae |  | 26 |
| Cryptarcha undata | Nitidulidae |  | 10 |
| Glischrochilus quadripunctatus | Nitidulidae |  | 3 |
| Cyanostolus aeneus | Monotomidae | NT | 1 |
| Rhizophagus bipustulatus | Monotomidae |  | 28 |
| Rhizophagus nitidulus | Monotomidae |  | 2 |
| Rhizophagus parvulus | Monotomidae |  | 6 |
| Silvanus bidentatus | Silvanidae |  | 1 |
| Cryptophagus micaceus | Cryptophagidae |  | 8 |
| Cryptophagus confusus | Cryptophagidae |  | 2 |
| Atomaria umbrina | Cryptophagidae |  | 2 |
| Atomaria subangulata | Cryptophagidae |  | 2 |
| Atomaria badia | Cryptophagidae |  | 1 |
| Atomaria bella | Cryptophagidae |  | 2 |
| Atomaria pulchra | Cryptophagidae |  | 3 |
| Tritoma bipustulata | Erotylidae |  | 2 |
| Triplax aenea | Erotylidae |  | 2 |
| Triplax russica | Erotylidae |  | 1 |
| Triplax rufipes | Erotylidae | NT | 1 |
| Dacne bipustulata | Erotylidae |  | 21 |
| Cerylon histeroides | Cerylonidae |  | 14 |
| Cerylon ferrugineum | Cerylonidae |  | 29 |
| Leiestes seminigra | Endomychidae | NT | 1 |
| Endomychus coccineus | Endomychidae |  | 3 |
| Latridius hirtus | Latridiidae |  | 4 |
| Enicmus fungicola | Latridiidae |  | 13 |
| Enicmus planipennis | Latridiidae | NT | 2 |
| Enicmus rugosus | Latridiidae |  | 24 |
| Enicmus testaceus | Latridiidae |  | 15 |
| Cis lineatocribratus | Cisidae |  | 1 |
| Cis alter | Cisidae |  | 1 |
| Cis jacquemarti | Cisidae |  | 1 |
| Cis glabratus | Cisidae |  | 1 |
| Cis comptus | Cisidae |  | 2 |
| Cis hispidus | Cisidae |  | 2 |
| Cis boleti | Cisidae |  | 6 |
| Cis rugulosus | Cisidae |  | 2 |
| Cis punctulatus | Cisidae |  | 1 |
| Ennearthron cornutum | Cisidae |  | 11 |
| Orthocis alni | Cisidae |  | 2 |
| Orthocis vestitus | Cisidae |  | 1 |
| Sulcacis affinis | Cisidae |  | 2 |
| Sulcacis fronticornis | Cisidae |  | 6 |
| Ropalodontus perforatus | Cisidae |  | 1 |
| Ropalodontus strandi | Cisidae |  | 2 |
| Octotemnus glabriculus | Cisidae |  | 4 |
| Synchita humeralis | Colydidae |  | 5 |
| Bitoma crenata | Colydidae |  | 3 |
| Litargus connexus | Mycetophagidae |  | 7 |
| Mycetophagus piceus | Mycetophagidae |  | 9 |
| Mycetophagus multipunctatus | Mycetophagidae |  | 2 |
| Mycetophagus fulvicollis | Mycetophagidae |  | 2 |
| Pyrochroa coccinea | Pyrochroidae |  | 6 |
| Schizotus pectinicornis | Pyrochroidae |  | 9 |
| Salpingus planirostris | Salpingidae |  | 9 |
| Salpingus ruficollis | Salpingidae |  | 4 |
| Euglenes pygmaeus/oculatus | Aderidae |  | 10 |
| Eledona agaricola | Tenebrionidae |  | 2 |
| Diaperis boleti | Tenebrionidae |  | 11 |
| Uloma culinaris | Tenebrionidae | NT | 1 |
| Corticeus fasciatus | Tenebrionidae | VU | 9 |
| Corticeus linearis | Tenebrionidae |  | 1 |
| Pseudocistela ceramboides | Tenebrionidae |  | 2 |
| Mycetochara flavipes | Tenebrionidae |  | 4 |
| Mycetochara humeralis | Tenebrionidae | NT | 4 |
| Mycetochara linearis | Tenebrionidae |  | 2 |
| Scraptia fuscula | Scraptidae |  | 12 |
| Anaspis frontalis | Scraptidae |  | 25 |
| Anaspis marginicollis | Scraptidae |  | 13 |
| Anaspis thoracica | Scraptidae |  | 31 |
| Anaspis rufilabris | Scraptidae |  | 24 |
| Anaspis flava | Scraptidae |  | 4 |
| Tomoxia bucephala | Mordellidae |  | 16 |
| Curtimorda maculosa | Mordellidae |  | 1 |
| Mordellistena variegata | Mordellidae | NT | 7 |
| Mordellistena humeralis | Mordellidae | NT | 7 |
| Orchesia undulata | Melandryidae |  | 15 |
| Phloiotrya rufipes | Melandryidae | NT | 2 |
| Hypulus quercinus | Melandryidae | NT | 4 |
| Conopalpus testaceus | Melandryidae |  | 1 |
| Rhagium sycophanta | Cerambycidae |  | 2 |
| Rhagium mordax | Cerambycidae |  | 12 |
| Alosterna tabacicolor | Cerambycidae |  | 13 |
| Anoplodera maculicornis | Cerambycidae |  | 5 |
| Leptura quadrifasciata | Cerambycidae |  | 4 |
| Phymatodes testaceus | Cerambycidae |  | 1 |
| Clytus arietis | Cerambycidae |  | 3 |
| Plagionotus arcuatus | Cerambycidae |  | 3 |
| Leiopus nebulosus | Cerambycidae |  | 1 |
| Stenostola dubia | Cerambycidae |  | 1 |
| Anthribus nebulosus | Anthribidae |  | 3 |
| Rhyncolus sculpturatus | Curculionidae |  | 2 |
| Hylesinus fraxini | Curculionidae |  | 2 |
| Scolytus laevis | Curculionidae |  | 1 |
| Scolytus intricatus | Curculionidae |  | 2 |
| Dryocoetes villosus | Curculionidae |  | 27 |
| Trypodendron domesticum | Curculionidae |  | 1 |
| Xyleborus dispar | Curculionidae |  | 14 |
| Xyleborus cryptophagus | Curculionidae |  | 4 |
| Xyleborinus saxesenii | Curculionidae |  | 20 |
| **Facultative saproxylic species** |  |  |  |
| Quedius invreai | Staphylinidae |  | 1 |
| Quedius scitus | Staphylinidae |  | 2 |
| Proteinus brachypterus | Staphylinidae |  | 7 |
| Phyllodrepa melanocephala | Staphylinidae |  | 1 |
| Coprophilus striatulus | Staphylinidae |  | 1 |
| Habrocerus capillaricornis | Staphylinidae |  | 1 |
| Lordithon thoracicus | Staphylinidae |  | 2 |
| Sepedophilus testaceus | Staphylinidae |  | 1 |
| Haploglossa gentilis | Staphylinidae |  | 2 |
| Microdota subtilis | Staphylinidae |  | 5 |
| Atheta harwoodi | Staphylinidae |  | 12 |
| Traumoecia picipes | Staphylinidae |  | 4 |
| Zyras cognatus | Staphylinidae |  | 2 |
| Gyrophaena affinis | Staphylinidae |  | 1 |
| Epuraea binotata | Nitidulidae |  | 2 |
| Epuraea aestiva | Nitidulidae |  | 1 |
| Latridius consimilis | Latridiidae |  | 3 |
| Latridius nidicola | Latridiidae |  | 3 |
| Stephostethus lardarius | Latridiidae |  | 2 |
| Stephostethus angusticollis | Latridiidae |  | 1 |
| Stephostethus pandellei | Latridiidae |  | 15 |
| Aridius nodifer | Latridiidae |  | 6 |
| Cartodere constricta | Latridiidae |  | 1 |
| Corticaria serrata | Latridiidae |  | 3 |
| Corticaria rubripes | Latridiidae |  | 1 |
| Corticaria longicollis | Latridiidae |  | 1 |
| Cortinicaria gibbosa | Latridiidae |  | 27 |
| Corticarina similata | Latridiidae |  | 1 |
| Corticarina fuscula | Latridiidae |  | 7 |
| Margarinotus spp. | Hiteridae |  | 27 |
| Agathidium varians | Leioddidae |  | 4 |
| Agathidium confusum | Leioddidae |  | 14 |
| Agathidium nigrinum | Leioddidae |  | 1 |
| Agathidium nigripenne | Leioddidae |  | 3 |
| Agathidium seminulum | Leioddidae |  | 9 |
| Agathidium badium | Leioddidae |  | 1 |
| Scydmaenus hellwigii | Scydmaenidae |  | 5 |
| Gabrius splendidulus | Staphylinidae |  | 22 |
| Bisnius fimetarius | Staphylinidae |  | 13 |
| Bisnius puella | Staphylinidae |  | 2 |
| Philonthus succicola | Staphylinidae |  | 16 |
| Philonthus addendus | Staphylinidae |  | 2 |
| Velleius dilatatus | Staphylinidae |  | 1 |
| Quedius mesomelinus | Staphylinidae |  | 37 |
| Quedius cruentus | Staphylinidae |  | 4 |
| Quedius brevis | Staphylinidae |  | 7 |
| Quedius xanthopus | Staphylinidae |  | 7 |
| Euplectus nanus | Staphylinidae |  | 10 |
| Euplectus piceus | Staphylinidae |  | 9 |
| Euplectus karstenii | Staphylinidae |  | 19 |
| Trimium brevicorne | Staphylinidae |  | 1 |
| Trichonyx sulcicollis | Staphylinidae |  | 2 |
| Acrulia inflata | Staphylinidae |  | 5 |
| Phyllodrepa nigra | Staphylinidae |  | 3 |
| Phyllodrepa gracilicornis | Staphylinidae |  | 2 |
| Omalium rivulare | Staphylinidae |  | 4 |
| Deliphrum tectum | Staphylinidae |  | 2 |
| Scaphisoma agaricnum | Staphylinidae |  | 12 |
| Scaphisoma boleti | Staphylinidae |  | 20 |
| Anotylus insecatus | Staphylinidae |  | 1 |
| Lordithon lunulatus | Staphylinidae |  | 18 |
| Sepedophilus littoreus | Staphylinidae |  | 8 |
| Sepedophilus immaculatus | Staphylinidae |  | 2 |
| Sepedophilus bipunctatus | Staphylinidae |  | 4 |
| Aleochara sparsa | Staphylinidae |  | 25 |
| Aleochara moerens | Staphylinidae |  | 5 |
| Oxypoda vittata | Staphylinidae |  | 1 |
| Oxypoda alternans | Staphylinidae |  | 10 |
| Thiasophila inquilina | Staphylinidae | NT | 3 |
| Haploglossa villosula | Staphylinidae |  | 5 |
| Atheta sodalis | Staphylinidae |  | 11 |
| Atheta gagatina | Staphylinidae |  | 10 |
| Atheta trinotata | Staphylinidae |  | 5 |
| Atheta crassicornis | Staphylinidae |  | 20 |
| Atheta euryptera | Staphylinidae |  | 5 |
| Atheta nigricornis | Staphylinidae |  | 38 |
| Atheta picipes | Staphylinidae |  | 4 |
| Anopleta corvina | Staphylinidae |  | 1 |
| Zyras lugens | Staphylinidae |  | 3 |
| Zyras laticollis | Staphylinidae |  | 1 |
| Bolitochara pulchra | Staphylinidae |  | 2 |
| Holobus flavicornis | Staphylinidae |  | 1 |
| Cetonia aurata | Scarabaidae |  | 2 |
| Anostirus castaneus | Elateridae |  | 1 |
| Aulonothroscus brevicollis | Throscidae |  | 1 |
| Megatoma undata | Dermestidae |  | 7 |
| Anthrenus museorum | Dermestidae |  | 2 |
| Ptinus fur | Anobiidae |  | 4 |
| Korynetes caeruleus | Cleridae |  | 1 |
| Carpophilus marginellus | Nitidulidae |  | 5 |
| Epuraea melanocephala | Nitidulidae |  | 3 |
| Epuraea placida | Nitidulidae |  | 14 |
| Epuraea terminalis | Nitidulidae |  | 3 |
| Epuraea variegata | Nitidulidae |  | 1 |
| Epuraea melina | Nitidulidae |  | 1 |
| Epuraea rufomarginata | Nitidulidae |  | 1 |
| Pocadius ferrugineus | Nitidulidae |  | 3 |
| Cychramus luteus | Nitidulidae |  | 8 |
| Glischrochilus hortensis | Nitidulidae |  | 29 |
| Sphindus dubius | Aspidiphoridae |  | 9 |
| Rhizophagus dispar | Monotomidae |  | 4 |
| Ahasverus advena | Silvanidae |  | 1 |
| Henoticus serratus | Cryptophagidae |  | 1 |
| Cryptophagus pubescens | Cryptophagidae |  | 1 |
| Cryptophagus dentatus | Cryptophagidae |  | 17 |
| Cryptophagus pallidus | Cryptophagidae |  | 4 |
| Cryptophagus pilosus | Cryptophagidae |  | 8 |
| Atomaria ornata | Cryptophagidae |  | 6 |
| Atomaria clavigera | Cryptophagidae |  | 2 |
| Atomaria fuscata | Cryptophagidae |  | 18 |
| Atomaria nigrirostris | Cryptophagidae |  | 13 |
| Atomaria atrata | Cryptophagidae |  | 4 |
| Atomaria procerula | Cryptophagidae |  | 1 |
| Palorus depressus | Tenebrionidae |  | 7 |
| Mordella holomelaena | Mordellidae |  | 4 |
