# Supplementary material for: Assemblages of saproxylic beetles on large downed trunks of oak
Source: Ecol Evol. 2016 Feb 12;6(6):1614–25. doi: 10.1002/ece3.1935 (PMC4751154; doi:10.1002/ece3.1935)
Supplement: Supplementary file 2 — Appendix S2. Saproxylic species included in the contrast between downed trunks of oaks and living veteran oaks. [file ECE3-6-1614-s002.docx]

**Appendix 2.** Saproxylic species included in the contrast between downed trunks of oaks and living veteran oaks. Number are odds ratios [ln(OR) with CI_95%_; positive values means a preference for downed trunks while negative means a preference for living trees. Species had been caught in 40 window traps on trees and 40 on downed trunks.

| Species | Family | Redlisted | ln(OR) | ±CI_95%_ |
| --- | --- | --- | --- | --- |
| **Obligate saproxylic species** |  |  |  |  |
| *Paromalus flavicornis/parallelepipedus* | Histeridae |  | 1.35 | 1.23 |
| *Plegaderus caesus* | Histeridae |  | 1.12 | 3.23 |
| *Stenichnus godarti* | Scydmaenidae |  | 2.02 | 3.00 |
| *Batrisodes delaporti* | Staphylinidae | VU | -1.12 | 3.23 |
| *Batrisodes venustus* | Staphylinidae |  | 1.12 | 3.23 |
| *Bibloporus bicolor* | Staphylinidae |  | 3.57 | 2.87 |
| *Bibloporus minutus* | Staphylinidae |  | 3.45 | 2.87 |
| *Euplectus bescidicus* | Staphylinidae |  | 2.02 | 3.00 |
| *Euplectus decipiens* | Staphylinidae |  | 1.66 | 3.07 |
| *Euplectus mutator* | Staphylinidae |  | 2.02 | 3.00 |
| *Euplectus punctatus* | Staphylinidae |  | 4.49 | 2.86 |
| *Hapalaraea pygmaea* | Staphylinidae |  | 0.46 | 1.35 |
| *Quedius brevicornis* | Staphylinidae |  | -1.12 | 3.23 |
| *Quedius maurus* | Staphylinidae |  | 1.12 | 3.23 |
| *Quedius plagiatus* | Staphylinidae |  | 1.66 | 3.07 |
| *Liocola marmorata* | Scarabaeidae |  | 0.96 | 1.43 |
| *Lucanus cervus* | Lucanidae |  | 1.12 | 3.23 |
| *Sinodendron cylindricum* | Lucanidae |  | 1.72 | 2.19 |
| *Ampedus balteatus* | Elateridae |  | 0.18 | 1.19 |
| *Ampedus cinnabarinus* | Elateridae | NT | -1.12 | 3.23 |
| *Ampedus hjorti* | Elateridae |  | -1.72 | 2.19 |
| *Ampedus nigroflavus* | Elateridae | NT | 2.02 | 3.00 |
| *Ampedus pomorum* | Elateridae |  | 3.26 | 2.08 |
| *Ampedus praeustus* | Elateridae |  | 2.53 | 2.93 |
| *Ampedus sanguineus* | Elateridae |  | 1.12 | 3.23 |
| *Calambus bipustulatus* | Elateridae | NT | -1.12 | 3.23 |
| *Cardiophorus ruficollis* | Elateridae |  | 1.12 | 3.23 |
| *Melanotus castanipes* | Elateridae |  | 0.78 | 1.46 |
| *Melanotus villosus* | Elateridae |  | -0.75 | 1.76 |
| *Procraerus tibialis* | Elateridae | NT | -2.02 | 3.00 |
| *Agrilus angustulus* | Buprestidae |  | -1.12 | 3.23 |
| *Agrilus biguttatus* | Buprestidae |  | 2.30 | 2.96 |
| *Agrilus laticornis* | Buprestidae | NT | -1.66 | 3.07 |
| *Agrilus sulcicollis* | Buprestidae |  | -0.43 | 1.85 |
| *Ctesias serra* | Dermestidae |  | -3.46 | 1.56 |
| *Globicornis emarginata* | Dermestidae |  | 1.12 | 3.23 |
| *Globicornis nigripes* | Dermestidae | NT | -2.56 | 2.11 |
| *Lyctus linearis* | Bostrichidae | VU | -0.72 | 2.44 |
| *Anobium nitidum* | Anobiidae |  | -2.73 | 2.91 |
| *Anobium rufipes* | Anobiidae |  | -1.15 | 2.31 |
| *Dorcatoma chrysomelina* | Anobiidae |  | -1.35 | 0.93 |
| *Dorcatoma dresdensis* | Anobiidae |  | 1.12 | 3.23 |
| *Dorcatoma flavicornis* | Anobiidae |  | -2.30 | 1.21 |
| *Gastrallus immarginatus* | Anobiidae |  | -2.43 | 1.56 |
| *Hedobia imperalis* | Anobiidae |  | -1.93 | 2.17 |
| *Microbregma emarginata* | Anobiidae |  | 1.66 | 3.07 |
| *Ptinus rufipes* | Anobiidae |  | 0.20 | 0.88 |
| *Ptinus sexpunctatus* | Anobiidae | NT | -1.12 | 3.23 |
| *Ptinus subpilosus* | Anobiidae |  | -2.46 | 1.13 |
| *Xestobium rufovillosum* | Anobiidae |  | 0.72 | 2.44 |
| *Xyletinus longitarsis* | Anobiidae | VU | 1.71 | 1.60 |
| *Xyletinus pectinatus* | Anobiidae |  | 1.12 | 3.23 |
| *Lymexylon navale* | Lymexylonidae |  | 1.33 | 1.07 |
| *Grynocharis oblonga* | Trogossitidae |  | -1.66 | 3.07 |
| *Tillus elongatus* | Cleridae |  | 1.12 | 3.23 |
| *Hypebaeus flavipes* | Malachidae |  | -2.56 | 2.11 |
| *Silvanus bidentatus* | Silvanidae |  | 1.12 | 3.23 |
| *Atomaria badia* | Cryptophagidae |  | 1.12 | 3.23 |
| *Atomaria bella* | Cryptophagidae |  | 1.66 | 3.07 |
| *Atomaria pulchra* | Cryptophagidae |  | 2.02 | 3.00 |
| *Atomaria subangulata* | Cryptophagidae |  | 1.66 | 3.07 |
| *Atomaria umbrina* | Cryptophagidae |  | 1.66 | 3.07 |
| *Cryptophagus badius* | Cryptophagidae |  | -1.12 | 3.23 |
| *Cryptophagus confusus* | Cryptophagidae |  | 1.66 | 3.07 |
| *Cryptophagus micaceus* | Cryptophagidae |  | -1.29 | 0.99 |
| *Dacne bipustulata* | Erotylidae |  | 2.30 | 1.21 |
| *Triplax aenea* | Erotylidae |  | 1.66 | 3.07 |
| *Triplax rufipes* | Erotylidae | NT | 1.12 | 3.23 |
| *Triplax russica* | Erotylidae |  | 0.00 | 2.81 |
| *Tritoma bipustulata* | Erotylidae |  | 1.66 | 3.07 |
| *Cerylon ferrugineum* | Cerylonidae |  | 3.48 | 1.37 |
| *Cerylon histeroides* | Cerylonidae |  | 1.58 | 1.22 |
| *Endomychus coccineus* | Endomychidae |  | 1.15 | 2.31 |
| *Leiestes seminigra* | Endomychidae | NT | 1.12 | 3.23 |
| *Cis alter* | Cisidae |  | 1.12 | 3.23 |
| *Cis boleti* | Cisidae |  | 2.73 | 2.91 |
| *Cis comptus* | Cisidae |  | 1.66 | 3.07 |
| *Cis fagi* | Cisidae |  | -1.12 | 3.23 |
| *Cis glabratus* | Cisidae |  | 1.12 | 3.23 |
| *Cis hispidus* | Cisidae |  | 1.66 | 3.07 |
| *Cis jacquemarti* | Cisidae |  | 1.12 | 3.23 |
| *Cis lineatocribratus* | Cisidae |  | 1.12 | 3.23 |
| *Cis punctulatus* | Cisidae |  | 1.12 | 3.23 |
| *Cis rugulosus* | Cisidae |  | 1.66 | 3.07 |
| *Ennearthron cornutum* | Cisidae |  | 2.69 | 2.10 |
| *Octotemnus glabriculus* | Cisidae |  | 2.30 | 2.96 |
| *Orthocis alni* | Cisidae |  | 1.66 | 3.07 |
| *Orthocis vestitus* | Cisidae |  | 1.12 | 3.23 |
| *Ropalodontus perforatus* | Cisidae |  | 1.12 | 3.23 |
| *Ropalodontus strandi* | Cisidae |  | 1.66 | 3.07 |
| *Sulcacis affinis* | Cisidae |  | 1.66 | 3.07 |
| *Sulcacis fronticornis* | Cisidae |  | 2.73 | 2.91 |
| *Litargus connexus* | Mycetophagidae |  | 2.90 | 2.90 |
| *Mycetophagus fulvicollis* | Mycetophagidae |  | 1.66 | 3.07 |
| *Mycetophagus multipunctatus* | Mycetophagidae |  | 0.72 | 2.44 |
| *Mycetophagus piceus* | Mycetophagidae |  | -1.64 | 0.98 |
| *Mycetophagus populi* | Mycetophagidae |  | -1.66 | 3.07 |
| *Euglenes pygmaeus/oculatus* | Aderidae |  | -1.37 | 0.94 |
| *Aderus populneus* | Aderidae | NT | -1.12 | 3.23 |
| *Allecula morio* | Tenebrionidae | NT | -3.90 | 2.86 |
| *Corticeus fasciatus* | Tenebrionidae | VU | 3.20 | 2.88 |
| *Corticeus linearis* | Tenebrionidae |  | 1.12 | 3.23 |
| *Diaperis boleti* | Tenebrionidae |  | 0.98 | 1.17 |
| *Eledona agaricola* | Tenebrionidae |  | -0.43 | 1.85 |
| *Mycetochara axillaris* | Tenebrionidae |  | -1.66 | 3.07 |
| *Mycetochara flavipes* | Tenebrionidae |  | 0.75 | 1.76 |
| *Mycetochara humeralis* | Tenebrionidae | NT | -0.81 | 1.29 |
| *Mycetochara linearis* | Tenebrionidae |  | -1.85 | 1.59 |
| *Pentaphyllus testaceus* | Tenebrionidae | NT | -2.53 | 2.93 |
| *Prionychus ater* | Tenebrionidae |  | -3.33 | 2.88 |
| *Pseudocistela ceramboides* | Tenebrionidae |  | -1.71 | 1.60 |
| *Uloma culinaris* | Tenebrionidae | NT | 1.12 | 3.23 |
| *Scraptia fuscula* | Scraptiidae |  | -1.47 | 0.94 |
| *Conopalpus testaceus* | Melandryidae |  | -2.56 | 2.11 |
| *Hypulus quercinus* | Melandryidae | NT | 2.30 | 2.96 |
| *Orchesia micans* | Melandryidae |  | -2.53 | 2.93 |
| *Orchesia undulata* | Melandryidae |  | 2.43 | 1.56 |
| *Phloiotrya rufipes* | Melandryidae | NT | 1.66 | 3.07 |
| *Alosterna tabacicolor* | Cerambycidae |  | 0.66 | 1.02 |
| *Anoplodera maculicornis* | Cerambycidae |  | 2.53 | 2.93 |
| *Clytus arietis* | Cerambycidae |  | 2.02 | 3.00 |
| *Grammoptera ustulata* | Cerambycidae |  | -2.02 | 3.00 |
| *Leiopus nebulosus* | Cerambycidae |  | -1.47 | 2.24 |
| *Leptura quadrifasciata* | Cerambycidae |  | 2.30 | 2.96 |
| *Phymatodes testaceus* | Cerambycidae |  | -1.93 | 2.17 |
| *Plagionotus arcuatus* | Cerambycidae |  | 2.02 | 3.00 |
| *Rhagium mordax* | Cerambycidae |  | 3.57 | 2.87 |
| *Rhagium sycophanta* | Cerambycidae |  | 0.72 | 2.44 |
| *Stenostola dubia* | Cerambycidae |  | 1.12 | 3.23 |
| *Rhyncolus sculpturatus* | Curculionidae |  | 1.66 | 3.07 |
| **Estimate for OBLIGATE** |  |  | **0.38** | **0.33** |
|  |  |  |  |  |
| **Facultative saproxylic species** |  |  |  |  |
| *Dendrophilus corticalis* | Histeridae |  | -2.02 | 3.00 |
| *Gnathoncus buyssoni/nannetensis* | Histeridae |  | -3.33 | 2.88 |
| *Margarinotus spp* | Histeridae |  | 3.04 | 1.23 |
| *Scydmaenus hellwigii* | Scydmaenidae |  | 2.53 | 2.93 |
| *Euplectus karstenii* | Staphylinidae |  | 3.56 | 2.08 |
| *Euplectus nanus* | Staphylinidae |  | 3.33 | 2.88 |
| *Euplectus piceus* | Staphylinidae |  | 3.20 | 2.88 |
| *Quedius brevis* | Staphylinidae |  | 2.28 | 2.13 |
| *Quedius cruentus* | Staphylinidae |  | 0.32 | 1.57 |
| *Quedius invreai* | Staphylinidae |  | -1.66 | 3.07 |
| *Quedius mesomelinus* | Staphylinidae |  | 4.39 | 1.46 |
| *Quedius scitus* | Staphylinidae |  | 1.66 | 3.07 |
| *Quedius xanthopus* | Staphylinidae |  | 1.56 | 1.62 |
| *Trichonyx sulcicollis* | Staphylinidae |  | 2.02 | 3.00 |
| *Trimium brevicorne* | Staphylinidae |  | 1.12 | 3.23 |
| *Velleius dilatatus* | Staphylinidae |  | -2.43 | 2.12 |
| *Trox scaber* | Trogidae |  | -1.66 | 3.07 |
| *Cetonia aurata* | Scarabaidae |  | 1.66 | 3.07 |
| *Anostirus castaneus* | Elateridae |  | 1.12 | 3.23 |
| *Aulonothroscus brevicollis* | Throscidae |  | -0.72 | 2.44 |
| *Anthrenus museorum* | Dermestidae |  | -1.98 | 1.58 |
| *Anthrenus scrophularie* | Dermestidae |  | -1.12 | 3.23 |
| *Attagenus pellio* | Dermestidae |  | -1.12 | 3.23 |
| *Dermestes lardarius* | Dermestidae |  | -1.12 | 3.23 |
| *Megatoma undata* | Dermestidae |  | -0.45 | 1.09 |
| *Ptinus fur* | Anobiidae |  | -0.81 | 1.29 |
| *Korynetes caeruleus* | Cleridae |  | 0.00 | 2.81 |
| *Ahasverus advena* | Silvanidae |  | 1.12 | 3.23 |
| *Atomaria atrata* | Cryptophagidae |  | 2.30 | 2.96 |
| *Atomaria clavigera* | Cryptophagidae |  | 1.66 | 3.07 |
| *Atomaria fuscata* | Cryptophagidae |  | 2.74 | 1.55 |
| *Atomaria morio* | Cryptophagidae |  | -2.73 | 2.91 |
| *Atomaria nigrirostris* | Cryptophagidae |  | 3.68 | 2.86 |
| *Atomaria ornata* | Cryptophagidae |  | 2.73 | 2.91 |
| *Atomaria procerula* | Cryptophagidae |  | 1.12 | 3.23 |
| *Cryptophagus dentatus* | Cryptophagidae |  | 1.89 | 1.21 |
| *Cryptophagus pallidus* | Cryptophagidae |  | 2.30 | 2.96 |
| *Cryptophagus pilosus* | Cryptophagidae |  | 1.56 | 1.62 |
| *Cryptophagus populi* | Cryptophagidae |  | -1.12 | 3.23 |
| *Cryptophagus pubescens* | Cryptophagidae |  | 1.12 | 3.23 |
| *Cryptophagus saginatus* | Cryptophagidae |  | -1.12 | 3.23 |
| *Cryptophagus scanicus* | Cryptophagidae |  | -4.39 | 2.86 |
| *Henoticus serratus* | Cryptophagidae |  | 1.12 | 3.23 |
| *Mycetophagus quadriguttatus* | Mycetophagidae | NT | -1.66 | 3.07 |
| *Palorus depressus* | Tenebrionidae |  | 2.11 | 2.15 |
| *Tenebrio molitor* | Tenebrionidae |  | -1.12 | 3.23 |
| **Estimate for FACULTATIVE** |  |  | **0.67** | **0.63** |
